# Small-angle X-ray scattering from GaN nanowires on Si(111): facet truncation rods, facet roughness and Porod’s law

**DOI:** 10.1107/S205327332001548X

**Published:** 2021-01-05

**Authors:** Vladimir M. Kaganer, Oleg V. Konovalov, Sergio Fernández-Garrido

**Affiliations:** a Paul-Drude-Institut für Festkörperelektronik, Leibniz-Institut im Forschungsverbund Berlin e. V., Hausvogteiplatz 5-7, 10117 Berlin, Germany; b European Synchrotron Radiation Facility, 71 avenue des Martyrs, 38043 Grenoble, France; c Grupo de electrónica y semiconductores, Departamento Física Aplicada, Universidad Autónoma de Madrid, C/ Francisco Tomás y Valiente 7, 28049 Madrid, Spain

**Keywords:** nanowires, Porod’s law, facet truncation rods, small-angle X-ray scattering, SAXS, grazing-incidence small-angle X-ray scattering, GISAXS

## Abstract

Small-angle X-ray scattering from GaN nanowires grown on Si(111) is measured in the grazing-incidence geometry and modelled by means of a Monte Carlo simulation that takes into account the orientational distribution of the faceted nanowires and the roughness of their side facets. It is found that the scattering intensity at large wavevectors does not follow Porod’s law *I*(*q*) ∝ *q* ^−4^. The intensity depends on the orientation of the side facets with respect to the incident X-ray beam. It is maximum when the scattering vector is directed along a facet normal, reminiscent of surface truncation rod scattering. At large wavevectors *q*, the scattering intensity is reduced by surface roughness. A root-mean-square roughness of 0.9 nm, which is the height of just 3–4 atomic steps per micrometre-long facet, already gives rise to a strong intensity reduction.

## Abstract 2

The intensity of small-angle X-ray scattering from GaN nanowires on Si(111) depends on the orientation of the side facets with respect to the incident beam. This reminiscence of truncation rod scattering gives rise to a deviation from Porod’s law. A roughness of just 3–4 atomic steps per micrometre-long side facet notably changes the intensity curves.

## 1. Introduction   

GaN nanowires (NWs) form spontaneously in plasma-assisted molecular-beam epitaxy (PA-MBE) on various substrates at elevated temperatures under an excess of N (Fernández-Garrido *et al.*, 2009 ▸, 2012 ▸). In contrast with the vapour–liquid–solid (VLS) growth approach followed to synthesize the majority of semiconductor NWs, PA-MBE growth of GaN NWs takes place without a metal particle on the top (Ristić *et al.*, 2008 ▸). The advantages of spontaneous formation are the absence of contamination from foreign metal particles and the possibility of fabricating axial heterostructures with sharp interfaces by alternating the supply of different elements.

GaN NWs on Si(111), which is the most common substrate, grow in dense ensembles (≳10^10^ cm^−2^) and initially possess radii of tens of nanometres as well as broad radius and length distributions (Consonni, 2013 ▸). As they grow in length, they bundle together (Kaganer *et al.*, 2016*a* ▸). In the process of bundling, two neighbouring NWs bend towards each other and merge their top parts, while the bottom parts remain separate. During further growth, the top parts gradually attain a hexagonal cross-sectional shape, so that in top-view images some of them may look like a single NW with a larger radius.

The long axes of GaN NWs are GaN[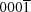] (Zúñiga-Pérez *et al.*, 2016 ▸). They are aligned on Si(111) in the surface normal direction, with a 3–5° wide distribution of the long-axis orientations (tilt) (Jenichen *et al.*, 2011 ▸). The side facets of the NWs, which are GaN(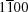) lattice planes (Brandt *et al.*, 2014 ▸), are epitaxially aligned with the Si(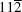) planes (Geelhaar *et al.*, 2011 ▸). The range of relative misorientation of these planes (twist) varies from 2 to 4° (Jenichen *et al.*, 2011 ▸).

For dense NW ensembles on Si(111), the radius distribution can be obtained from the analysis of top-view scanning electron micrographs (Brandt *et al.*, 2014 ▸). However, this method provides the radius distribution of only the top parts of the NWs, which notably differs from the radius distribution in their bottom parts because of NW bundling. In addition, the use of scanning electron micrographs for statistical analysis of NW radii becomes much more laborious for NW ensembles with low densities such as those formed on TiN (van Treeck *et al.*, 2018 ▸), since when the magnification of the scanning electron micrographs is chosen to quantify the NW diameters, only a few NWs fall into the field of view.

Small-angle X-ray scattering (SAXS) is potentially more suitable than scanning electron microscopy for the determination of the radius distribution of GaN NW ensembles grown on Si(111) because it probes the entire NW volume. From the standpoint of small-angle scattering, GaN NWs are long hexagonal prisms with a substantial distribution of their cross-sectional sizes and orientations. Since these NWs are, on average, aligned along the substrate surface normal, the incident X-ray beam must be directed at a grazing incidence to the substrate surface. Grazing-incidence small-angle X-ray scattering (GISAXS) has been employed to study Si (David *et al.*, 2008 ▸; Buttard *et al.*, 2013 ▸), GaAs (Mariager *et al.*, 2007 ▸) and InAs (Eymery *et al.*, 2007 ▸, 2009 ▸; Mariager *et al.*, 2009 ▸) NWs grown by the VLS growth mechanism with Au nanoparticles on their tops. Unlike spontaneously formed GaN NWs, NW ensembles prepared by VLS are characterized by very narrow distributions of the NW sizes and orientations. The scattering intensity from such NW ensembles possesses the same features as the scattering intensity from a single NW: it exhibits oscillations due to the interference caused by reflections at opposite facets and a pronounced dependence of the intensity on the scattering vector orientation. The scattering intensity has maxima in the facet normal directions, which are referred to as facet truncation rods, and these are well established in X-ray diffraction from nanoparticles (Renaud *et al.*, 2009 ▸) and stem from crystal truncation rods from planar surfaces (Robinson, 1986 ▸; Robinson & Tweet, 1992 ▸).

In the case of GaN NWs, despite the potential advantages of GISAXS to assess the distribution of NW radii, we are not aware of any GISAXS study to date. The closest report is the work of Horák *et al.* (2008 ▸), who performed an in-plane X-ray diffraction study of GaN NWs using a laboratory diffractometer. Their analysis implies the absence of strain in the NWs. If so, the NW diameters can be obtained from ω/2θ scans in the same way as can be done in GISAXS. However, this analysis cannot be applied to dense arrays of GaN NWs, which are inhomogeneously strained as a result of NW bundling (Jenichen *et al.*, 2011 ▸; Kaganer *et al.*, 2012 ▸; Fernández-Garrido *et al.*, 2014 ▸; Kaganer *et al.*, 2016*b* ▸). We do not discuss here other X-ray diffraction studies of NWs devoted to the determination of strain and composition since they are beyond the scope of the present work.

The aim of the present paper is to develop the approaches required for the analysis of GaN NW arrays by GISAXS using dense NW ensembles grown on Si(111) as a model example. Since GaN NWs are faceted crystals (their side facets are 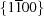 planes), we expected that the GISAXS intensity at large wavevectors would follow Porod’s law. Porod’s law (Porod, 1951 ▸; Debye *et al.*, 1957 ▸) states that, at large wavevectors *q*, the small-angle scattering intensity *I*(*q*) from particles with sharp boundaries (*i.e.* possessing an abrupt change in the electron density at the surface) follows a universal asymptotic law *I*(*q*) ∝ *q* ^−4^. Sinha *et al.* (1988 ▸) pointed out a common origin of Porod’s law in small-angle scattering and Fresnel’s law for reflection from flat surfaces, namely that the scattering intensity from a planar surface in the *xy* plane is proportional to 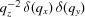. An average over random orientations of the plane gives rise to the *q* ^−4^ law just because the delta function δ(*q*) has a dimensionality of *q* ^−1^. Sinha *et al.* (1988 ▸) performed an explicit calculation of the orientational average. Deviations from Porod’s law are caused by fractality or the roughness of the surfaces in porous media (Bale & Schmidt, 1984 ▸; Wong & Bray, 1988 ▸; Sinha, 1989 ▸).

In this paper, we show that the GISAXS intensity from GaN NWs at large wavevectors depends on the azimuthal orientation of the NW ensemble with respect to the incident X-ray beam. The intensity is maximum when the scattering vector is directed along the facet normal, and minimum when the scattering vector is parallel to the facet. In other words, the azimuthal dependence of the GISAXS intensity reveals the facet truncation rods. We also show that the intensity at large *q* reveals the roughness of the side facets of the GaN NWs. We determine a root-mean-squared (r.m.s.) roughness of about 0.9 nm, corresponding to the height of a few atomic steps on a micrometre-long NW sidewall facet. Both the facet truncation rod scattering and the surface roughness cause deviations from Porod’s law.

## 2. Experiment   

For the present study, we selected three samples, here numbered 1 to 3, with different NW lengths from the series A studied by Kaganer *et al.* (2016*a* ▸). The GaN NWs were synthesized in an MBE system equipped with a solid-source effusion cell for Ga and a radio-frequency N_2_ plasma source for generating active N. The samples were grown on Si(111) substrates, which were preliminarily etched in dilute HF (5%), outgassed above 1173 K for 30 min to remove any residual Si_*x*_O_*y*_ from the surface, and exposed to the N plasma for 10 min. The substrate growth temperature was approximately 1073 K, as measured with an optical pyrometer. The Ga and N fluxes, calibrated by determining the thickness of GaN films grown under N- and Ga-rich conditions (Heying *et al.*, 2000 ▸), were 0.3 and 0.75 monolayers per second, respectively. The growth time is the only parameter that was varied among the samples to obtain ensembles of NWs with different lengths.

**Figure 1:**
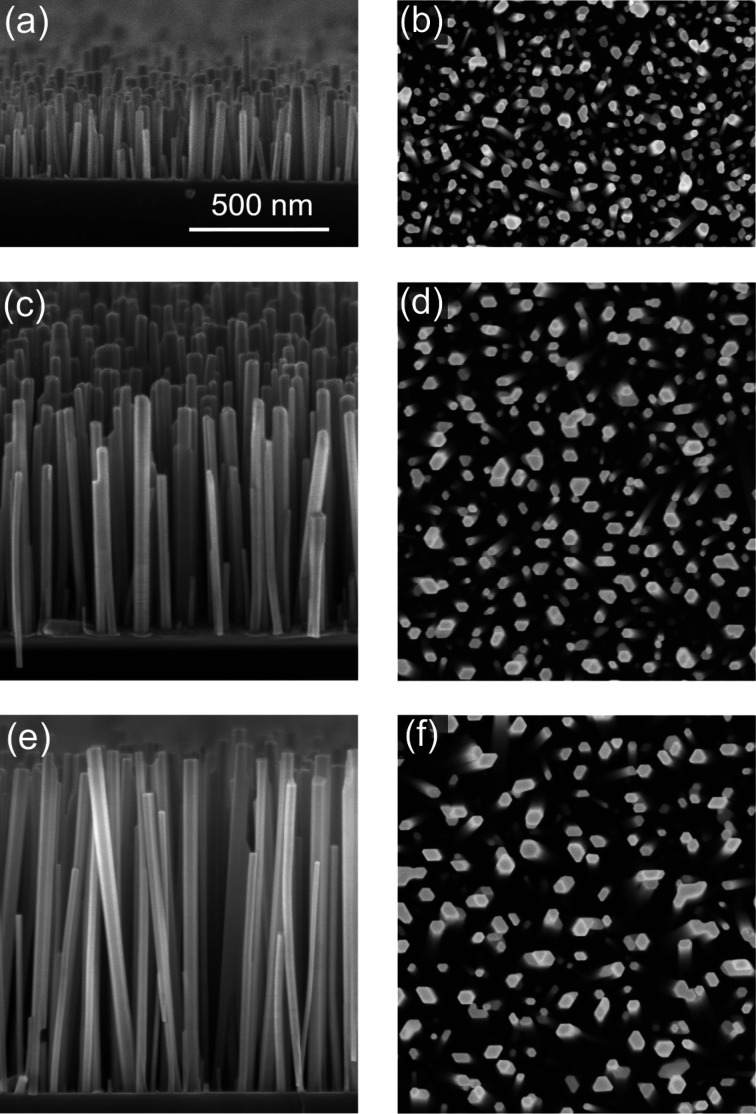
(Left) Bird’s-eye-view and (right) top-view scanning electron micrographs of (*a*), (*b*) sample 1, (*c*), (*d*) sample 2 and (*e*), (*f*) sample 3. The average NW lengths are 230, 650 and 985 nm, respectively. The scale bar in panel (*a*) is applicable to all the micrographs.

Fig. 1 ▸ presents scanning electron micrographs of samples 1–3. Sample 1 corresponds to the end of the NW nucleation process. The NW density is 3.5 × 10^10^ cm^−2^, while the average length and diameter of the NWs are 230 and 22 nm, respectively. The NWs are mostly uncoalesced hexagonal prisms. Samples 2 and 3 display the further growth of the NWs, with average NW lengths of 650 nm for sample 2 and 985 nm for sample 3. The average NW diameter, as determined by the analysis of the top-view micrographs (right-hand column of Fig. 1 ▸), increases with increasing NW length, while the NW density decreases.

The increase in diameter is a result of NW bundling, rather than their radial growth. A decisive proof of the absence of radial growth comes from the measurement of the fraction of the total area that is covered by NWs (Kaganer *et al.*, 2016*a* ▸). The area fraction covered by NWs, derived from the top-view micrographs shown in the right-hand column of Fig. 1 ▸, does not change from one sample to another and remains always at 20%. The bundling is clearly seen in Figs. 1 ▸(*c*) and 1 ▸(*e*). The NWs remain thin (and possess larger density) in their bottom parts and merge in their top parts. By merging, they reduce the surface energy of side facets at the cost of bending energy (Kaganer *et al.*, 2016*a* ▸). The bending energy may be reduced further by the introduction of dislocations at the merging joints (Kaganer *et al.*, 2016*b* ▸). The NW segments are then less bent and have kinks at the joints.

The distribution of the NW orientations was determined with a laboratory X-ray diffractometer. We measured the full width at half-maximum (FWHM) of the GaN(0002) reflection to determine the tilt range with respect to the substrate surface normal, and the GaN(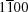) reflection to determine the twist range with respect to the in-plane orientation of the substrate. The FWHM of the tilt distribution is found to decrease with increasing NW length, from 5.1° for sample 1 to 4.0° and 3.9° for samples 2 and 3, respectively, as a consequence of bundling. The FWHMs of the twist distribution are 2.8°, 2.7° and 3.1° for samples 1, 2 and 3, respectively.

The GISAXS measurements were performed on the beamline ID10 at the European Synchrotron Radiation Facility (ESRF) using an X-ray energy of 22 keV (wavelength λ = 0.5636 Å). The incident beam was directed at grazing incidence to the substrate. The chosen grazing-incidence angle was 0.2°, *i.e.* about 2.5 times larger than the critical angle of the substrate, to avoid possible complications of the scattering pattern typical for grazing-incidence X-ray scattering (Renaud *et al.*, 2009 ▸). A two-dimensional Pilatus 300K detector (Dectris) placed at a distance of 2.38 m from the sample provided a resolution of 8.06 × 10^−3^ nm^−1^.

**Figure 2:**
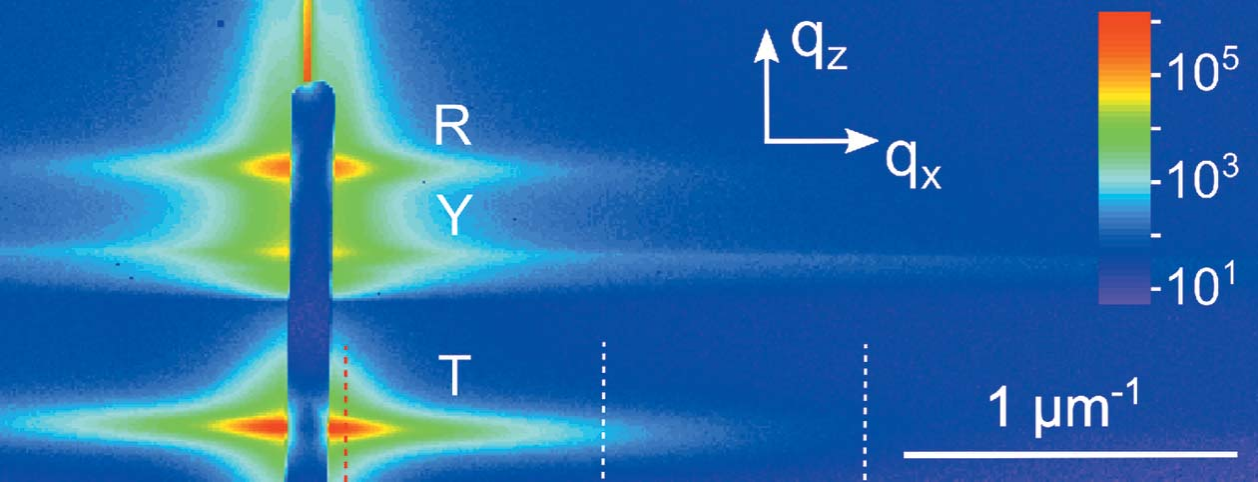
GISAXS intensity from sample 1 as measured by a two-dimensional detector. The scattering around the transmitted beam, the scattering around the beam reflected from the substrate surface and the Yoneda streak are labelled T, R and Y, respectively. The vertical blue bar in the middle of the scattering pattern is the beamstop. The three vertical dashed lines mark the positions of the scans presented in Fig. 3. The colour-coded scale bar represents the intensity in counts.

Fig. 2 ▸ shows the GISAXS intensity measured from sample 1. The scattering pattern comprises three horizontal streaks. The small-angle scattering around the transmitted beam is labelled ‘T’, while the scattering around the beam reflected from the substrate surface is labelled ‘R’. Both streaks reveal the same scattering intensity dependence on the lateral wavevector *q*_*x*_. The scattering around the transmitted beam possesses a larger intensity. For that reason, the T streak is chosen here for further analysis. Besides the T and R streaks, the intensity distribution in Fig. 2 ▸ contains the Yoneda streak, marked ‘Y’, which is located at the critical angle for total external reflection. The chosen incidence angle of 0.2° allows us to separate the three different streaks well, which facilitates the analysis of the GISAXS intensity within the framework of kinematic scattering.

## 3. Analysis of the measured intensities   

We use the specific features of the NWs as oriented long prisms to improve the accuracy of the determination of the GISAXS intensity *I*(*q*_*x*_) from the measured maps. Since a single NW is a needle-like object, its scattering intensity in reciprocal space is concentrated in the plane perpendicular to the long axis of the NW. A random tilt of a NW results in a corresponding tilt of the intensity plane. Hence, one can expect that the spread of 4–5° in the directions of the long axes of the NWs results in a sector of intensity in Fig. 2 ▸, with the width Δ*q*_*z*_ increasing proportional to *q*_*x*_.

**Figure 3:**
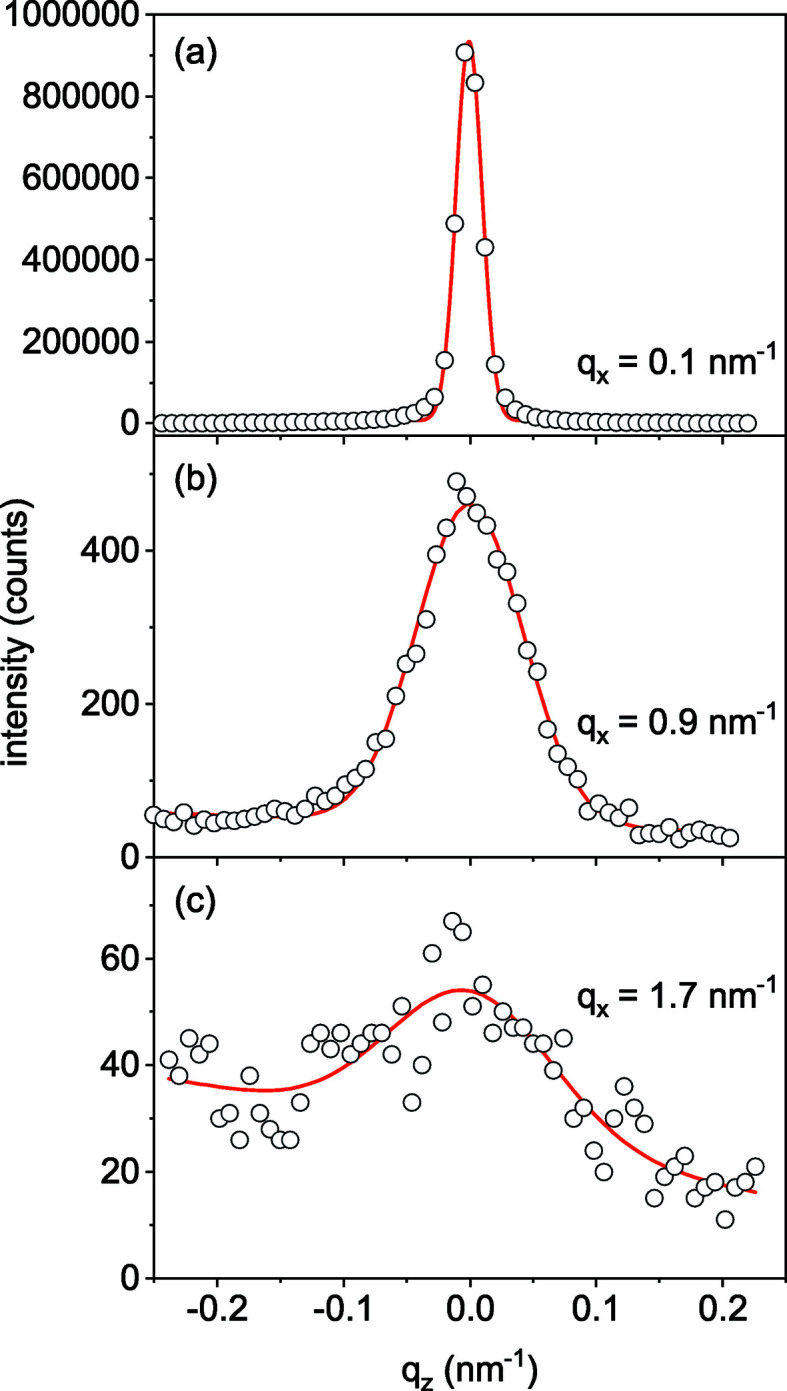
Measured intensity profiles (circles) along the lines of constant *q*_*x*_ marked by the dashed lines in Fig. 2 ▸, and the respective Gaussian fits (lines).

**Figure 4:**
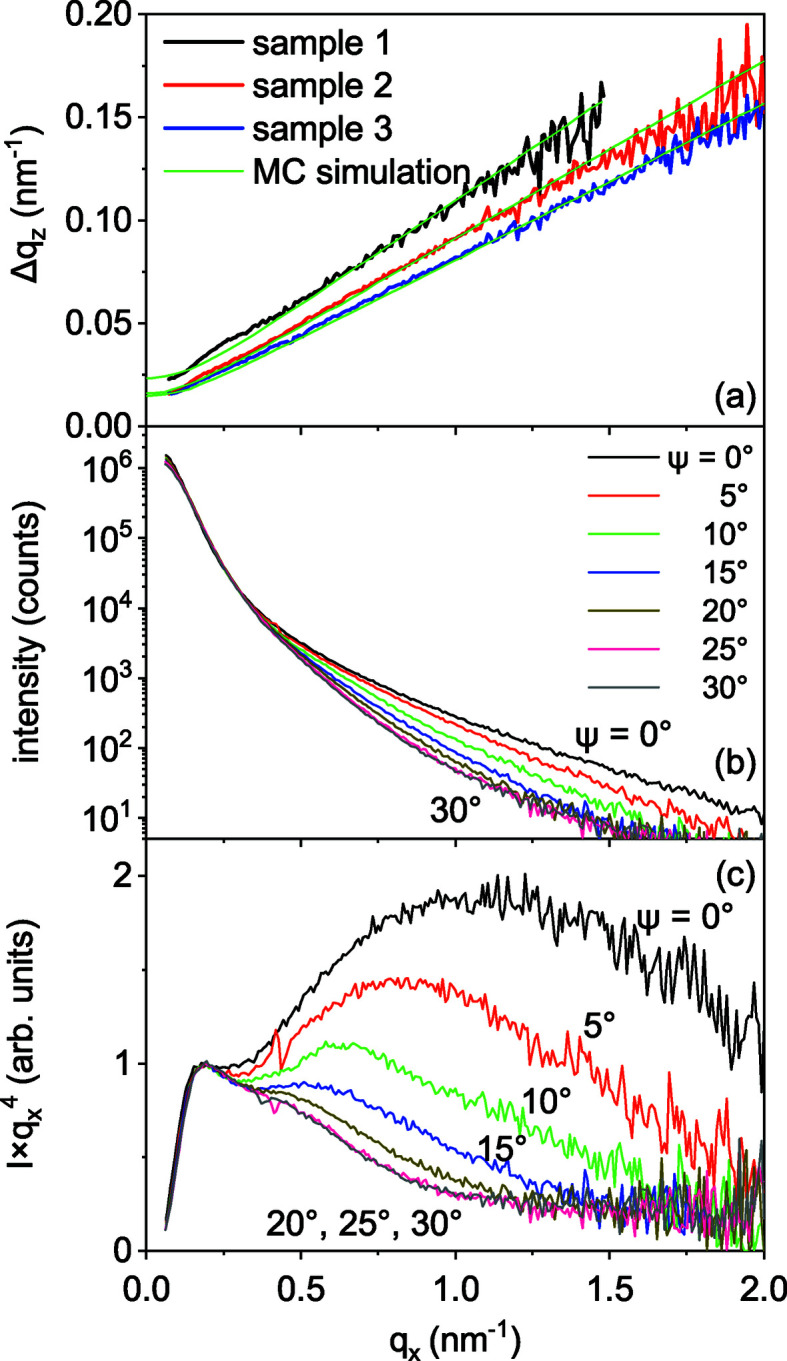
(*a*) FWHMs of the intensity profiles Δ*q*_*z*_ as a function of the wavevector *q*_*x*_. Green lines are the results of the Monte Carlo simulations described in Section 4. (*b*) GISAXS intensity of sample 1 as a function of the wavevector *q*_*x*_ at varying azimuthal orientations ψ. (*c*) The same intensity profiles as in panel (*b*) but plotted as 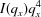 versus *q*_*x*_.

Fig. 3 ▸ presents intensity profiles along the dotted lines indicated in Fig. 2 ▸, *i.e.* scans at constant values of *q*_*x*_. These profiles are fitted by a Gaussian plus a background that may linearly depend on *q*_*z*_. The FWHMs of these profiles Δ*q*_*z*_ are plotted in Fig. 4 ▸(*a*). As expected, Δ*q*_*z*_ increases linearly with *q*_*x*_. One can also see that the straight lines through the data points for each sample do not pass through the origin. The additional broadening can be attributed to the effect of the finite NW length *L*, so that, in the first approximation, the intersection point at *q*_*x*_ = 0 is Δ*q*_*z*_ = 2π/*L*. For a more accurate determination of the NW lengths, we performed Monte Carlo simulations of the Δ*q*_*z*_ versus *q*_*x*_ curves. The Monte Carlo simulations are described below in Section 4, and the parameters obtained in the simulations are presented in Table 1 ▸. The results of the simulations are shown in Fig. 4 ▸(*a*) by green lines.

The angular ranges of the NW orientations are 5.9°, 5.1° and 4.6° for samples 1, 2 and 3, respectively. They can be obtained from the slopes Δ*q*_*z*_/*q*_*x*_ of the respective curves in Fig. 4 ▸(*a*). These values are close to (albeit somewhat larger than) the widths of the orientational distributions measured by Bragg diffraction, as described in Section 2.

The average NW length of 230 nm for sample 1 obtained in the Monte Carlo simulation coincides with the length that can be obtained from the scanning electron micrograph in Fig. 1 ▸(*a*). The average lengths for samples 2 and 3 are 350 and 400 nm, respectively, notably smaller than the NW lengths in Figs. 1 ▸(*c*) and 1 ▸(*e*). The difference can be explained by NW bundling. The NW length measured in the GISAXS experiment is not its total length but an effective length of the NW segment between the merging joints. From the ratio of the total NW length to the segment length (see Table 1 ▸), we find that a NW consists of 2–3 segments.

The fits in Fig. 3 ▸ help to improve the determination of the scattering intensity *I*(*q*_*x*_) at both small and large momenta *q*_*x*_. At small *q*_*x*_, the intensity profiles are narrow and the peak intensity has to be determined from just a few data points. At large *q*_*x*_, the intensity is low and the background is comparable to the signal. After performing the fits of the cross-sectional profiles (*i.e.* along the *q*_*z*_ direction) shown in Fig. 3 ▸ and establishing the linear dependence of the FWHM Δ*q*_*z*_ on *q*_*x*_, we make one more step to reduce the noise of the data. Linear fits are made for the Δ*q*_*z*_ on *q*_*x*_ dependencies plotted in Fig. 4 ▸(*a*). Then, the fits of the *q*_*z*_ profiles shown in Fig. 3 ▸ are repeated, now with the FWHMs fixed at the values obtained from the linear fits. In this way, the number of free parameters in the Gaussian fits is decreased and the curves *I*(*q*_*x*_) are smoothed. These curves are used in the subsequent analysis.

Fig. 4 ▸(*b*) presents the GISAXS intensity *I*(*q*_*x*_) measured on sample 1 at varying azimuthal orientation ψ. The sample orientation ψ = 0 corresponds to the incident X-ray beam along a GaN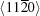 direction, so that the scattering vector (the *x*-axis direction) is along 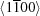, which is the normal to the NW facets. Fig. 4 ▸(*b*) comprises the measurements obtained on the rotation of sample 1 about the normal to the substrate surface (*i.e.* about the direction of the long axes of the NWs) from ψ = 0° to 30° with a step of 5°. Since the sample has a rectangular shape and the illuminated area varies on rotation, the curves are scaled to obtain the same intensity in the small-*q*_*x*_ range. The scaling factors differ by a factor of less than 2. The azimuthal dependence of the intensity at large *q*_*x*_ is evident from the plot.

In the case of the reflected beam (streak R in Fig. 2 ▸), an identical analysis of the intensity (not shown here) results in curves close to those shown in Fig. 4 ▸(*b*). Thus, we observe the same azimuthal dependence of the intensity but with a smaller total intensity and a higher level of noise. For this reason, for the further analysis presented here we exclusively consider the intensity distributions around the transmitted beam.

Since we expect Porod’s law *I*(*q*_*x*_) ∝ 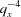 to be satisfied at large *q*_*x*_, we plot in Fig. 4 ▸(*c*) the same data as 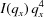 versus *q*_*x*_, which would tend to a constant value for large *q*_*x*_. Surprisingly, a strong deviation from Porod’s law is observed. Furthermore, the data not only deviate from Porod’s law, but also exhibit a strong azimuthal dependence. In order to explain this un­expected behaviour, in the next section we develop a Monte Carlo method to calculate the scattering intensity.

## 4. Calculation of the scattering intensity   

### 4.1. Scattering amplitude of a prism   

We calculate first the scattering amplitude (form factor) of a NW *A*(**q**) in a coordinate system linked to the NW, *i.e.* with the *z* axis in the direction of the long axis of the NW. Hence, the cross section of the NW is in the *xy* plane. Next, we will consider in Section 4.3 a transformation of the wavevectors from the laboratory frame to the NW coordinate system, and perform an average of the intensities |*A*(**q**)|^2^ over different NW orientations.

The scattering amplitude of a NW is given by its form factor 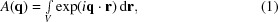
where the integral is calculated over the NW volume *V*. Since the NW is a prism, the scattering amplitude can be represented as a product of the components along the NW axis and in the plane perpendicular to it, *A*(**q**) = *A*_∥_(*q*_∥_)*A*_⊥_(**q_⊥_**). The longitudinal component is simply 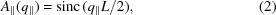
where 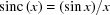 and *L* is the NW length.

The calculation of the transverse component *A*_⊥_(**q_⊥_**) can be reduced to a sum over the vertices, as was initially shown for faceted crystals by von Laue (1936 ▸) and used nowadays to calculate the form factors of nanoparticles (Vartanyants *et al.*, 2008 ▸; Renaud *et al.*, 2009 ▸; Pospelov *et al.*, 2020 ▸). Specifically, von Laue (1936 ▸) proposed to reduce, using the Gauss theorem, the volume integral (1) to the integrals over the facets; application of the Gauss theorem to these area integrals reduces them to integrals over the edges, which, in turn, can be taken by parts and expressed through the coordinates of the vertices.

**Figure 5:**
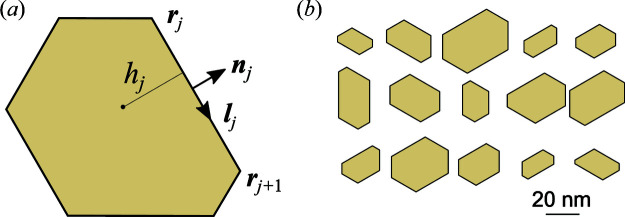
(*a*) A hexagon with vertices **r**_*j*_ and the unit vectors along and normal to the side **l**_*j*_ and **n**_*j*_. The distance from the hexagon centre to its side is *h*_*j*_. (*b*) Examples of randomly generated hexagons used to simulate the scattering from sample 1.

For a planar polygon, the form factor reads
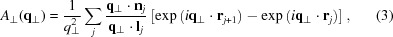
where the sum runs over the vertices and, as illustrated in Fig. 5 ▸(*a*), **r**_*j*_ are coordinates of the vertices, and **l**_*j*_ and **n**_*j*_ are unit vectors along the polygon side between the vertices **r**_*j*_ and **r**_*j*+1_ and normal to it, respectively. Lee & Mittra (1983 ▸) proposed another expression for the form factor, 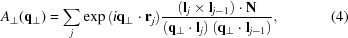
where **N** is the unit vector normal to the polygon plane, and Wuttke (2017 ▸) explicitly showed the identity of expressions (3) and (4). Equation (3) makes it possible to resolve easily the numerical uncertainty 0/0 that arises at **q**_⊥_ · **l**_*j*_ = 0. Since **l**_*j*_ = (**r**_*j*+1_ − **r**_*j*_)/|**r**_*j*+1_ − **r**_*j*_|, we have in the limit **q**_⊥_ · **l**_*j*_ → 0 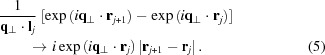



**Figure 6:**
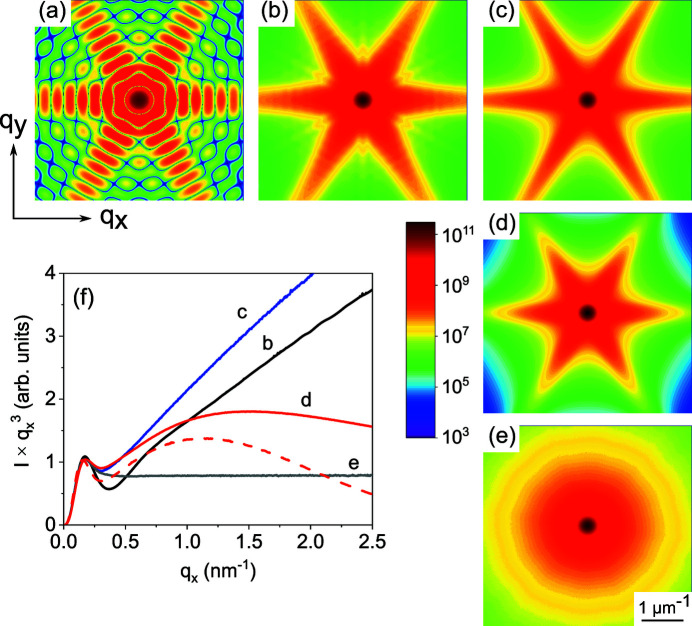
Scattering intensities from (*a*) a regular hexagon with a side length of 12 nm, (*b*) a distribution of regular hexagons with an average side length of 12 nm and a standard deviation of the side lengths of 4 nm, (*c*) a distribution of distorted hexagons as shown in Fig. 5 ▸(*b*), (*d*) the same distribution as in panel (*c*) but with a side facet roughness σ = 0.6 nm added according to equation (7), and (*e*) a distribution of randomly oriented distorted hexagons. The colour-coded scale bar representing the intensity is applicable to panels (*a*)–(*e*). (*f*) Radial intensity distributions from panels (*b*)–(*e*) in the directions of the intensity maxima; the product *Iq* ^3^ is plotted. The curves are labelled by the same letters as the respective maps. The effect of roughness is illustrated in (*f*) by two curves: the solid red curve corresponds to the geometric distribution of the atomic steps on the side facets and the dashed red curve to the Poisson distribution, both possessing the same r.m.s. roughness of σ = 0.6 nm.

Fig. 6 ▸(*a*) shows the intensity distribution calculated by equation (3) for a regular hexagon with a side length of 12 nm. The intensity is higher in the directions of the side normals and oscillates due to interference from opposite sides of the hexagon. Fig. 6 ▸(*b*) shows a Monte Carlo calculation of the average intensity from hexagons of different sizes. A log-normal distribution of the lengths of the hexagon sides is taken with the same mean value of 12 nm and a standard deviation of 4 nm. This choice of parameters corresponds approximately to the respective values for sample 1.

The radial intensity distribution in the direction along the intensity maximum is presented in Fig. 6 ▸(*f*) by the black line. The intensity distribution is presented as the product 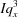, which would be constant at large *q*_*x*_ for an ensemble of randomly oriented hexagons (as well as for other two-dimensional objects with rigid boundaries) after averaging over all possible orientations. As stated above, the intensity maxima in Fig. 6 ▸(*b*) correspond to the directions normal to the sides of the hexagon. They possess, at large *q*_*x*_, an 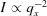 dependence due to a steplike variation in the density at a planar surface. Hence, in Fig. 6 ▸(*f*), we observe a linear increase in the intensity for large values of *q*_*x*_ (black line).

The local maximum at *q*_m_ = 0.175 nm^−1^ in Fig. 6 ▸(*f*) is related to the mean length of the side facet of the hexagons *a* = 12 nm as *a* ≃ 2.1/*q*_m_, which allows us to determine the hexagon size directly from plots of 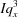 versus *q*_*x*_ (in the three-dimensional case of hexagonal prisms, the same formula is applicable for the maximum in an 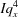 versus *q*_*x*_ plot, see Section 4.3). For comparison, the form factor of a circle of radius *R* gives 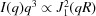, where *J*_1_(*x*) is the Bessel function. The first maximum of *J*_1_(*x*) at *x* ≃ 1.84 gives the circle radius *R* ≃ 1.84/*q*_m_. We can relate a hexagon and a circle even closer, by defining an effective radius *R*_*a*_ of a circle possessing the same area as a hexagon with a side length *a*. Then, we have *R*_*a*_ = 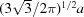 ≃ 0.91*a* and *R*_*a*_ ≃ 1.9/*q*_m_, with the proportionality coefficient very close to the case of a circle.

With the form factor defined by the positions of the vertices according to either equation (3) or (4), we are not restricted to regular hexagons but can take into account the real cross-sectional shapes of the NWs. Since the side facets of the NWs are GaN{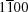} planes making an angle of 60° to each other, we build the hexagons as shown in Fig. 5 ▸(*a*): random heights *h*_*j*_ are taken in the directions normal to the facets. Then, we check that the generated hexagon is convex, and discard it otherwise. Fig. 5 ▸(*b*) presents examples of randomly generated hexagons with the same orientation of their sides. The distribution of the hexagon shapes is chosen to simulate sample 1 and is further described in Section 5. The intensity map obtained from this distribution of hexagons is shown in Fig. 6 ▸(*c*). The respective radial intensity distribution is presented in Fig. 6 ▸(*f*) as the blue line. One can see that the black and blue lines in Fig. 6 ▸(*f*) are remarkably different. In particular, the hexagon shape distribution notably reduces the dip in intensity. Therefore, the distortion of the hexagons can be deduced from the intensity plots.

### 4.2. Roughness of the side facets   

The side facets of GaN NWs are atomically flat (Stoica *et al.*, 2008 ▸; Ristić *et al.*, 2008 ▸) but may have atomic steps. The radial growth of these NWs presumably proceeds by step flow, with the motion of steps from the NW top, where they are nucleated, down along the side facets (Fernández-Garrido *et al.*, 2013 ▸). Random steps across the side facets can be treated as facet roughness in the same way as is done in the calculation of crystal truncation rods (Robinson, 1986 ▸; Robinson & Tweet, 1992 ▸).

A step of height *d*_0_ shifts the *j*th side of the polygon in Fig. 5 ▸ by a vector *d*_0_ **n**_*j*_ in the direction of the facet normal. Hence, the *j*th term in sum (3) acquires an additional factor 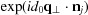. Random steps give rise to a factor *R*_*j*_ = *R*(**q**_⊥_ · **n**_*j*_), where the function *R*(*q*) is defined as 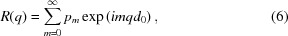
and *p*_*m*_ are the probabilities of the shift of the side facet by *m* steps. Hence, the function *R*(*q*) is the characteristic function of the probabilities *p*_*m*_.

Consider the geometric probability distribution *p*_*m*_ = (1 − β)β^*m*^ with the parameter β < 1. It describes a flat surface with a fraction β one step higher, its fraction β is, in turn, one step higher, and so on (Robinson, 1986 ▸). The r.m.s. roughness is σ = *d*_0_(β^1/2^)/(1 − β) and the corresponding characteristic function is 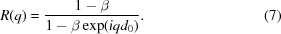



The Poisson probability distribution 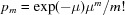 gives rise to the r.m.s. roughness σ = *d*_0_(μ^1/2^) and the characteristic function is 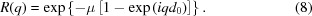



We stress here that the *j*th term in sum (3) is multiplied by a complex factor *R*_*j*_ = *R*(**q**_⊥_ · **n**_*j*_) that depends on the orientation of the respective facet. This is different from a common treatment of surface roughness, which involves a single factor |*R*|^2^. In particular, the Poisson probability distribution gives for 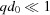 the factor |*R*|^2^ = 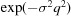. Buttard *et al.* (2013 ▸) used such a factor to describe the effect of roughness on the scattered intensity from Si NWs, by analogy with the roughness of planar surfaces, and arrived at an r.m.s. roughness σ of 1 nm for their samples. We use equation (3) in further calculations with the complex factors *R*_*j*_ in each term of the sum.

Fig. 6 ▸(*d*) shows the scattering intensity distribution obtained with the roughness factors given by equation (7). The r.m.s. roughness is taken to be σ = 0.6 nm and the step height *d*_0_ is that of the atomic steps on the GaN(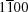) facet, *d*_0_ = *a*_0_ 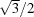, where *a*_0_ = 0.319 nm is the GaN lattice spacing. Strictly speaking, the roughness factors given by equations (7) or (8) are derived for a prism which has in each cross section a hexagon with straight sides. It describes a variation in the cross section of the prism along its length and does not make sense for two-dimensional objects. Hence, the intensity distribution shown in Fig. 6 ▸(*d*) corresponds to prisms with perfectly aligned long axes.

The solid red line in Fig. 6 ▸(*f*) shows the radial intensity distribution obtained from the map shown in Fig. 6 ▸(*d*) in the direction of maximum intensity, calculated using the roughness factors for the geometric probability distribution given by equation (7). The dashed red line in Fig. 6 ▸(*f*) shows the intensity from the same distribution of hexagons but calculated using the roughness factors derived from the Poisson probability distribution, equation (8). The r.m.s. roughness is taken to be the same in both cases, σ = 0.6 nm. One can see that the roughness qualitatively changes the intensity at *q*σ > 1. Hence, the r.m.s. roughness σ can be obtained from the intensity plots. Moreover, the intensity curves are fairly sensitive to the choice of the probability distribution. Crystal truncation rods from planar surfaces possess a similar sensitivity to the choice of the roughness model (Walko, 2000 ▸). Our modelling of the scattering from GaN NWs presented in Section 5 shows that the geometric probability distribution provides a better agreement with the experimental data.

### 4.3. Orientational distribution of NWs   

The scattering intensity is measured as a function of the wavevector **q** in the laboratory frame (see Fig. 2 ▸). We need to find the components of this vector in the frame given by the long axis of the NW and the normal to one of its side facets. Let us consider first the simple case of the two-dimensional rotation of the hexagons (or perfectly aligned prisms in the plane normal to their long axes). The unit vector normal to a hexagon side (or prism facet) can be written as 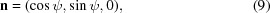
where ψ is an azimuthal angle (defined modulo 60°) with respect to a reference orientation. The unit vector along the hexagon side is 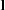 = 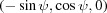. The components *q*_*n*_, *q*_*l*_ of the two-dimensional vector **q**_⊥_ in the plane perpendicular to the long axis of the NW are determined simply as *q*_*n*_ = **q_⊥_** · **n** and *q*_*l*_ = **q_⊥_** · **l**.

Fig. 6 ▸(*e*) presents a Monte Carlo calculation of the intensity for the distribution of distorted hexagons described above and sketched in Fig. 5 ▸(*b*), after averaging over the orientations ψ uniformly distributed from 0° to 360°. The corresponding radial intensity distribution, shown in Fig. 6 ▸(*f*) by a grey line, follows the two-dimensional Porod law *I*(*q*) ∝ *q* ^−3^ at large *q*. At small *q*, it coincides with the intensity distribution for the oriented hexagons.

**Figure 7:**
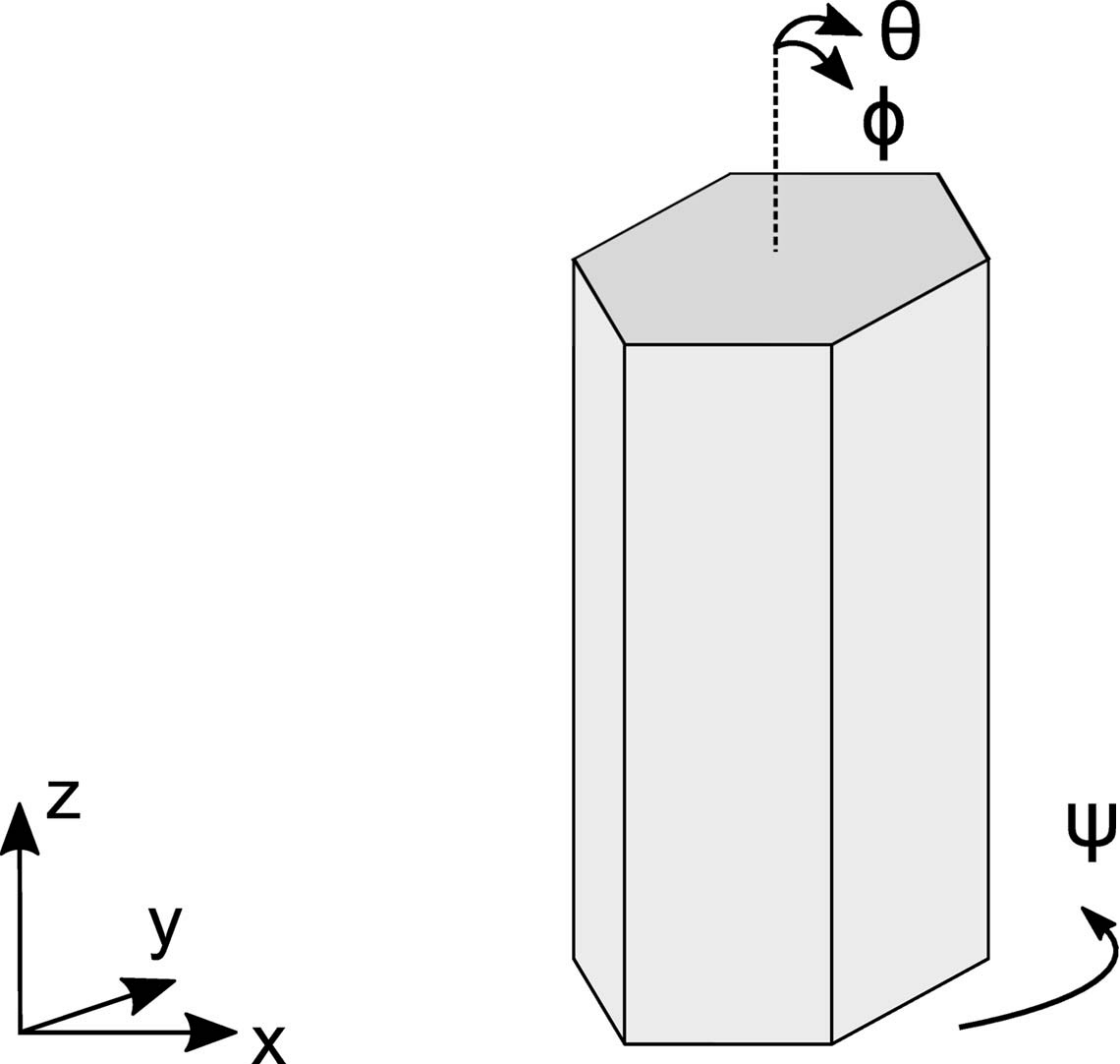
Three-dimensional rotation of a NW. The angles θ, ϕ and ψ are the angles of rotations about the *x*, *y* and *z* axes, respectively, and referred to as roll, pitch and yaw angles in nautical notation. The angles θ and ϕ define a random orientation of the long axis of the NW and vary in the range 3–5°, as determined from the tilt measurements. The mean value of the angle ψ defines an average orientation of the side facets with respect to the incident X-ray beam, and its variation in the range 2–4° is determined from the twist measurements.

For the three-dimensional distribution of the NW orientations, inherent to the spontaneous formation of GaN NWs on Si(111) (see Fig. 1 ▸), we obtain the components of the scattering vector **q**′ for a given NW by applying three rotations about the orthogonal axes *x*, *y* and *z* to the scattering vector **q** in the laboratory frame, **q**′ = *M*_*z*_(ψ)*M*_*y*_(ϕ)*M*_*x*_(θ)**q**. Here the scattering vector in the laboratory frame is shown in Fig. 2 ▸: the *q*_*x*_ axis is normal to the mean direction of the long axes of the NWs, the *q*_*z*_ axis is along this direction and *q*_*y*_ = 0. The matrices *M*_*j*_ (*j* = 1, 3) are the rotation matrices about the respective axes, and the angles θ, ϕ and ψ defining the NW orientation are the nautical angles, as shown in Fig. 7 ▸: the roll and pitch angles θ and ϕ define the direction of the long axis of the NW, and the yaw angle ψ the orientation of the side facets.

If the NW orientations are completely random, *i.e.* the angles θ, ϕ and ψ vary uniformly and independently from 0 to 2π, the small-angle scattering intensity at *q* ≫ 2π/*a*, where *a* is the width of the side facet, follows Porod’s law *I*(*q*) ∝ *q* ^−4^. However, since the NWs are long prisms, the scattering intensity from a single NW of length *L* with its long axis in the *z* direction concentrates in reciprocal space in a disc of width Δ*q*_*z*_ = 2π/*L*. We have seen in Section 3 that the scattering from oriented NWs is limited by Δ*q*_*z*_/*q*_*x*_ < Δθ, where Δθ is the angular range of orientations. As long as 2π/(*Lq*_*x*_) < Δθ, oriented NWs give the same intensity in the *x* direction as fully randomly oriented ones. Therefore, Porod’s law is satisfied for *q*_*x*_ > 2π/(*L*Δθ).

**Figure 8:**
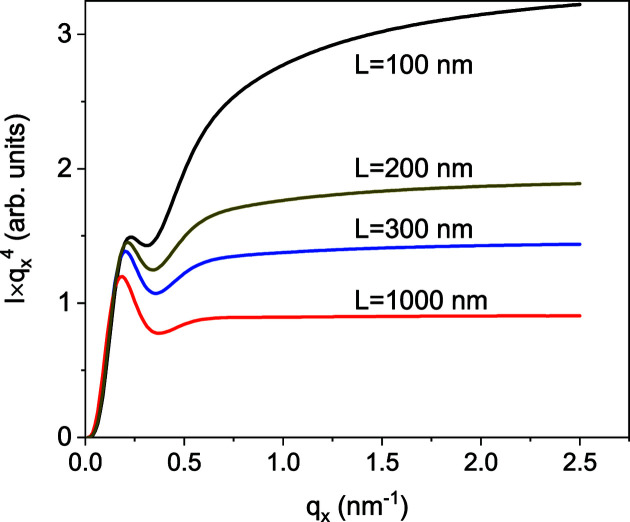
Monte Carlo calculation of the small-angle scattering intensity from an ensemble of NWs with a 5° wide range of orientations of the long axes and random orientation of the side facets. The width of the side facets is 12 nm and the NW lengths vary from 100 to 1000 nm.

Fig. 8 ▸ presents Monte Carlo calculations of the small-angle scattering intensity from NWs of different lengths and the same FWHMs of the distributions of the angles θ and ϕ of 5° corresponding to that of sample 1. In particular, for NWs with a length *L* = 200 nm, the condition derived above reads *q*_*x*_ > 0.7 nm^−1^, which is in good agreement with the region of constant 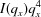 in Fig. 8 ▸. Hence, the limited range of tilt angles in the NW ensemble does not prevent the system reaching Porod’s law, even for the relatively short NWs of sample 1. The curves in Fig. 8 ▸ are calculated by averaging over the facet orientation ψ varying from 0 to 2π. Further Monte Carlo calculations, taking into account the orientational ordering of the side facets of the epitaxially grown GaN NWs, are presented in the next section.

## 5. Results   

**Figure 9:**
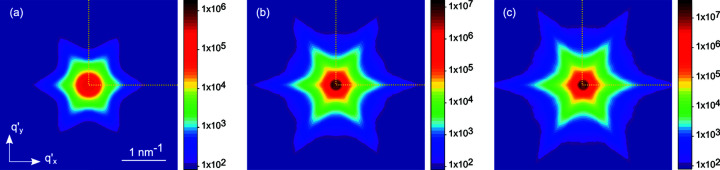
Scattering intensities in the plane normal to the mean direction of the long axes of the NWs for (*a*) sample 1, (*b*) sample 2 and (*c*) sample 3. The maps are obtained from the measurements *I*(*q*_*x*_) at different azimuthal orientations ψ and presented with 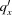 = 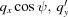 = 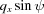. The measurements are performed in the quadrant 0 < ψ < π/2, shown by the dotted lines, and then reflected relative to the horizontal and vertical axes. The scale bars present the intensity in counts.

Figs. 9 ▸ and 10 ▸ present the results of systematic GISAXS measurements on samples 1–3. The measurements and their analysis are described in Sections 2 and 3, respectively. The samples were measured with the azimuthal orientation ψ varying from 0° to 90° in steps of 5°. Each measurement provided a map 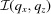 around the transmitted beam similar to the one in Fig. 2 ▸. The intensity 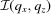 has been analysed by fitting every scan of a constant *q*_*x*_ by a Gaussian, as shown in Fig. 3 ▸. The peak values of the *q*_*z*_ scans obtained in this fit provided the intensity *I*(*q*_*x*_).

The measurements performed at different azimuthal orientations ψ of the sample are presented in Fig. 9 ▸ as maps in the axes 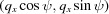. The measurements were performed in the quadrant 0 < ψ < π/2, shown by dotted lines, and then reflected relative to the horizontal and vertical axes. A sixfold symmetry of the intensity distribution, with maxima in the directions of the normals to the side facets of the NWs, is evident from the figure.

**Figure 10:**
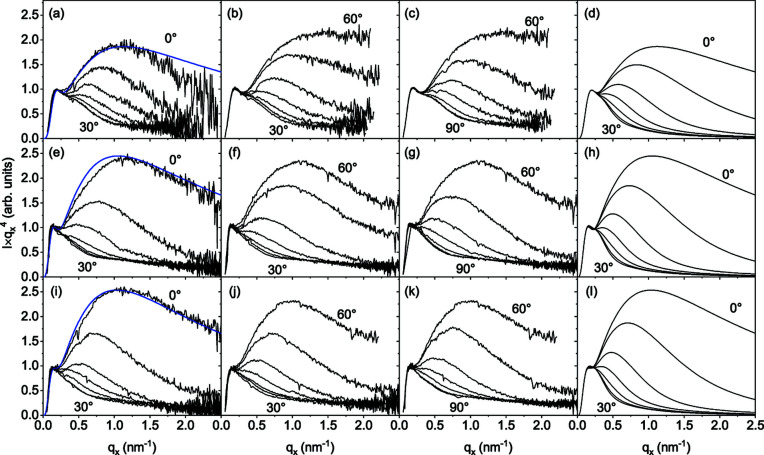
The measured GISAXS intensities (three left-hand columns) and Monte Carlo simulations (right-most column) for (*a*)–(*d*) sample 1, (*e*)–(*h*) sample 2 and (*i*)–(*l*) sample 3. The measurements are performed for different mean orientation angles ψ of the side facets of the NWs with respect to the X-ray beam, namely from 0° to 90° in steps of 5°. For clarity, these measurements are presented in three different panels (from 0° to 30°, from 30° to 60° and from 60° to 90°). The intensities are plotted as 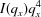 versus *q*_*x*_ to highlight deviations from Porod’s law. The curves calculated at ψ = 0° for each sample are repeated as blue curves in the left-most column, for direct comparison of the calculated and measured curves.

The same data are presented in Fig. 10 ▸ for each azimuthal angle ψ as a product 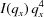 versus *q*_*x*_, to reveal deviations from Porod’s law. The data are represented in more detail: one can see that the sixfold symmetry is quantitatively proven for samples 2 and 3, while sample 1 reveals some difference between the curves at ψ = 0° and 60°. This difference is presumably due to the illumination of different parts of the sample during its rotation.

We calculate the scattering intensity by the Monte Carlo method. It enables a simultaneous integration over the distributions of the NW lengths, their cross-sectional sizes and shapes, and the orientations of both the NW long axis and of the side facets. The calculations take a fairly short time. It takes less than a minute on a single CPU core of a standard PC to calculate an intensity curve to an accuracy sufficient to make estimates. The smooth curves presented in this paper took less than an hour of CPU time each.

Table 1 ▸ summarizes the parameters of the NW ensembles obtained from the experiment and used as input for the Monte Carlo simulations, as well as the ones derived from the Monte Carlo simulations. We have simulated the *q*_*z*_ scans, similar to the ones presented in Fig. 3 ▸, and obtained their FWHMs Δ*q*_*z*_ as a function of *q*_*x*_. The results of these calculations are presented in Fig. 4 ▸(*a*) as green lines. For sample 1, agreement between the measurements and the simulations is found at a mean NW length of 230 nm, coinciding with the mean NW lengths in the scanning electron micrograph in Fig. 1 ▸(*a*). For samples 2 and 3, the simulations of the Δ*q*_*z*_ versus *q*_*x*_ curves give mean NW lengths of 350 and 400 nm, respectively, values notably smaller than the real NW lengths in Figs. 1 ▸(*c*) and 1 ▸(*e*). This difference is explained, as we have already discussed in Section 3, by NW bundling: the lengths obtained in the GISAXS analysis are the effective lengths of the NW segments between the merging joints of the bundled NWs. These NW lengths are used in further Monte Carlo simulations. The NW length distributions are assumed to be log-normal with a standard deviation of 20% from their respective average lengths.

Taking into account the sixfold symmetry of the intensity distributions in Fig. 9 ▸, we performed Monte Carlo simulations for the azimuth ψ from 0° to 30°. We assumed a normal distribution of the angle ψ, with the FWHM determined by the in-plane X-ray diffraction scans (see Table 1 ▸).

The parameters of the NW ensemble to be determined from the Monte Carlo simulations are the mean width of the side facets *a*, its variation and the variation in the cross-sectional shapes of the NWs, and the roughness of the side facets. We have seen in Section 3 that these parameters affect the calculated curves in qualitatively different ways. The mean facet size *a* determines the position of the local maximum of the curves at *q*_*x*_ ≃ 0.17 nm^−1^, which corresponds to a side facet width of about 12 nm. The depth of the dip between this maximum and the rise of the curves at larger *q*_*x*_ is controlled by the width of the facet size distribution and the shape distribution of the cross sections. The decrease in 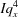 at large *q*_*x*_ is caused by the roughness of the side facets.

The distorted cross sections of the NWs were modelled in the Monte Carlo study as described in Section 4.1. The heights *h*_*j*_ shown in Fig. 5 ▸(*a*) were generated at random around a mean value. Fig. 5 ▸(*b*) exemplifies the shapes of the NWs used in the simulation of sample 1. The right-most column in Fig. 10 ▸ presents the Monte Carlo calculations of the small-angle scattering intensity for samples 1–3. For a direct comparison of the calculated and measured intensities, the curves calculated for each sample at ψ = 0° are repeated as blue lines in the left-most column of the figure.

**Figure 11:**
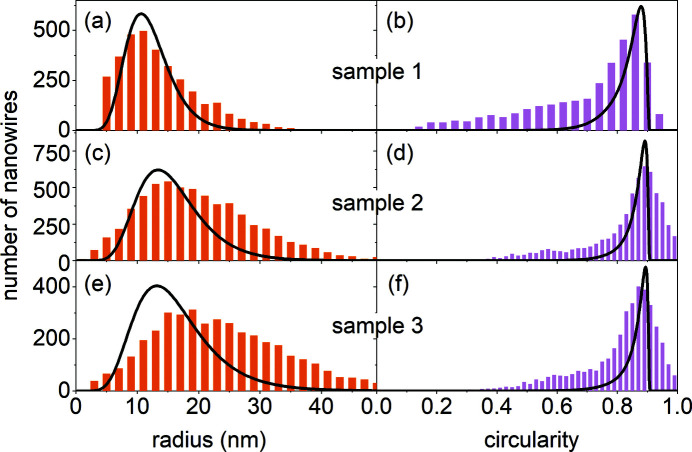
Distributions of the NW radii and circularities of samples 1–3 obtained from analysis of the top-view scanning electron micrographs (histograms) and the Monte Carlo modelling (lines).

For each generated NW, we calculate the cross-sectional area *A* and the perimeter *P*. Then, we determine out of these parameters the radius *R* from *A* = π*R* ^2^ and the circularity *C* = 4π*A*/*P* ^2^. The circularity thus defined is *C* = 1 for a circle, *C* = 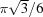 ≃ 0.907 for a regular hexagon, and 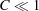 for highly irregular shapes. These parameters, radius and circularity, can be obtained from scanning electron micrographs and are objective NW shape descriptors as discussed elsewhere (Brandt *et al.*, 2014 ▸). The lines in Fig. 11 ▸ show the distributions of the radius and the circularity obtained in the simulations.

The mean NW radii, the standard deviations of the radius distributions and the roughnesses obtained in the Monte Carlo simulations for samples 1–3 are presented in Table 1 ▸. The simulations show the fairly high sensitivity of the calculated curves to these parameters. The accuracy of the determination of the mean radius and the standard deviation of the radius distribution can be estimated as ± 0.3 nm, while the r.m.s. roughness is determined with an accuracy of ± 0.02 nm. From the simulations in Fig. 3 ▸(*a*), we estimate the accuracy of the determination of the tilt angle distribution to be ± 0.1°, while the accuracy of the NW length distribution can be estimated as ± 50 nm.

The distributions of the cross-sectional radii and circularities of the NW ensembles were also interdependently obtained by analysing top-view scanning electron micrographs similar to those shown in Figs. 1 ▸(*d*)–1 ▸(*f*). The analysis was performed using the open-source software *ImageJ* (Schneider *et al.*, 2012 ▸), as described in detail by Kaganer *et al.* (2016*a* ▸) in their supporting information. The distribution of the radius obtained from the modelling of the GISAXS intensity for sample 1 in Fig. 11 ▸(*a*) is fairly close to the distribution derived from the micrographs. The circularity distribution obtained from the micrographs is, however, extended towards smaller values, indicating a higher density of NWs with elongated cross-sectional shapes. Such a discrepancy can be attributed to an artefact caused by the NW tilt. Specifically, the scanning electron micrographs exhibit very little difference in brightness between the top facet and the top part of the side facet of the NW, so that *ImageJ* treats both regions together, *i.e.* as extended intensity spots.

In contrast with sample 1, the NW radii obtained from the Monte Carlo simulations of the scattering intensity from samples 2 and 3 [Figs. 11 ▸(*c*) and 11 ▸(*e*)] are smaller than those derived from analysis of the scanning electron micrographs, and the discrepancy increases with increasing NW length. We remind the reader that the mean NW radius can be derived directly from the position *q*_*x*_ of the local maximum in the experimental curves presented in Fig. 10 ▸. It remains at about *q*_*x*_ ≃ 0.17 nm^−1^ and shifts only slightly to smaller values (and hence to larger radii) as the NW length increases from sample 1 to sample 3.

The origin of the discrepancy between the NW radii determined from the scanning electron micrographs and from the modelling of the GISAXS intensity is in the bundling of the NWs. Bundling is almost absent for sample 1, and the cross sections of the NWs obtained from the micrographs characterize the NWs along their full length. As the NWs grow in length, they bundle together, which causes an apparent radial growth. Simultaneously, the NW density decreases, so that the fraction of the surface covered by the NWs remains constant (Kaganer *et al.*, 2016*a* ▸). GISAXS provides statistics of the NW radii averaged over their lengths, while the top-view micrographs reveal their distribution at the top. The result is a progressive difference between the distributions obtained by the two methods.

The widths of the circularity distributions in the right-hand column of Fig. 11 ▸ reduce slightly with the growth of the NWs. The NW images in the scanning electron micrographs become more circular since, during NW growth, the bundled NWs attain a common shape that tends to a regular hexagon. Also, the low-circularity wing of the circularity histogram reduces, because the effective radii of the bundled NWs increase, and the distinction between the top facets and the top parts of the side facets becomes more pronounced for the *ImageJ* analysis. The circularity distributions obtained from the GISAXS intensity curves are sharper than the ones obtained from scanning electron micrographs, because the former take into account both single NWs in their bottom part and bundled NWs in their top part, while the latter count only the NW tops. We also remember that the circularity of a distorted hexagon is always smaller than the circularity *C* ≃ 0.907 of a regular hexagon. Larger circularities obtained from analysis of the scanning electron micrographs in Figs. 11 ▸(*d*) and 11 ▸(*f*) are due to the finite resolution of the micrographs, as well as to the algorithm used by *ImageJ* that tends to round faceted objects.

We have seen in Section 4.1 and particularly in Fig. 6 ▸(*f*) that, when the scattering vector is oriented normal to the side facets of the NWs (ψ = 0°) and the facets are atomically flat, facet truncation rod scattering would result in a linear increase in the intensity on the 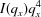 versus *q*_*x*_ plot at large *q*_*x*_. The decrease in the experimental curves indicates a roughness of the side facets. We obtain in the Monte Carlo modelling r.m.s. roughnesses of σ = 0.9, 0.95 and 0.85 nm for samples 1, 2 and 3, respectively. According to the height of the atomic steps on a GaN(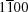) facet *d*_0_ = *a*_0_ 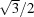 = 0.276 nm (here *a*_0_ = 0.319 nm is the GaN lattice spacing), the r.m.s. roughness is less than 3.5 steps.

## 6. Discussion and summary   

GaN NWs nucleate spontaneously on Si(111) and grow with a substantial disorder with respect to their orientations. Their growth is, nevertheless, epitaxial: the NWs inherit the out-of-plane and in-plane orientations of the substrate. Since these NWs are typically long (from hundreds of nanometres to a few micrometres) and thin (tens of nanometres), the range of orientations of their long axes of 3–5° is sufficient to provide the same average in the small-angle scattering intensity as if they would have all orientations in space. However, an angular range of orientations of the side facets of 3° gives rise to features in the GISAXS intensity distribution that are reminiscent of the crystal truncation rod scattering from flat surfaces of single crystals.

We have found that the GISAXS intensity depends on the orientation of the side facets with respect to the incident X-ray beam direction. In our experiment, the incident beam is kept normal to the average direction of the long axes of the NWs. The orientation of the incident beam with respect to the side facets is varied by rotating the sample about the substrate surface normal. The scattering intensity is maximum when the incident beam is along the facets, or in other words, when the scattering vector is in the direction of the facet normal.

The X-ray scattering intensity from a planar surface is proportional to *q* ^−2^. Porod’s law (*q* ^−4^) is a result of a full average over all orientations of the plane (Sinha *et al.*, 1988 ▸), *i.e.* integration over the two angles defining the plane orientation. For GaN NWs on Si(111), the range of orientations of the long axes is large enough to provide an integration over the tilt angle and give rise to a *q* ^−3^ dependence when the scattering vector is along the facet normal. In the *Iq* ^4^ versus *q* plots shown in Fig. 10 ▸, this dependence is seen as an intensity increase after the dip at ψ = 0° or 60°. The intensity decreases as the sample is rotated about the normal to the substrate surface. The minimum intensity value is reached at ψ = 30°, *i.e.* in the direction between facets.

The surface roughness gives rise to a decrease in the intensity at *q*σ ≳ 1, where σ is the r.m.s. roughness. The Monte Carlo modelling of the experimental curves in Fig. 10 ▸ gives σ from 0.85 to 0.95 nm, which is just 3.5 times the height of the atomic steps. Nevertheless, this small roughness strongly modifies the intensity curve for high values of *q*.

The GISAXS curves vary fairly little from one sample to another, despite the large difference between the cross-sectional sizes of the NWs observed in the scanning electron micrographs shown in Fig. 1 ▸. This apparent discrepancy is explained by NW bundling, which is an essential effect in their growth (Kaganer *et al.*, 2016*a* ▸). While GISAXS reflects the distribution of the cross-sectional sizes of the NWs over their whole volume, the top-view micrographs shown in the right-hand column of Fig. 1 ▸ reveal the cross-sectional sizes of the NWs at their very top part. As a result, the distributions of the NW radii and circularities obtained from the scanning electron micrographs and the GISAXS intensity curves only coincide for sample 1, which is free of bundling. As the NWs grow in height and their bundling increases (samples 2 and 3), the discrepancies between the results obtained by these two different methods increase.

Finally, we conclude that GISAXS, together with Monte Carlo modelling of the intensity curves, is well suited for the determination of the distributions of the cross-sectional sizes of NWs. The methods developed in the present paper are not specific to GaN NWs on Si(111) and can be applied to other NW distributions and material systems. In particular, they will be applied in a separate study to assess the radius distributions of GaN NW ensembles grown on TiN, which exhibit a much lower density that hinders analysis of the NW cross-sectional shapes by scanning electron microscopy.

## Figures and Tables

**Table 1:** Parameters of the NW ensembles The NW lengths obtained from the scanning electron microscopy (SEM) images and the FWHMs of the tilt distributions obtained by X-ray diffraction (XRD) are compared with their respective values obtained by the Monte Carlo (MC) simulations presented in Figs. 4 ▸(*a*) and 10 ▸. The FWHMs of the twist distributions are obtained by XRD and used in the Monte Carlo simulations. The NW radii (mean and standard deviation, st. dev.) and roughnesses are obtained by the Monte Carlo simulations of the GISAXS intensity curves.

	NW length (nm)		FWHM tilt (°)		FWHM twist (°)	NW radius (MC) (nm)		Roughness
	SEM	MC	XRD	MC	XRD	Mean	St. dev.	(MC) (nm)
Sample 1	230	230	5.1	5.9	2.8	12.1	3.8	0.9
Sample 2	650	350	4.0	5.1	2.7	15.8	5.6	0.95
Sample 3	985	400	3.9	4.6	3.1	16.3	6.5	0.95
